# Morphometric Study of the Hard Palate and Its Relevance to Dental and Forensic Sciences

**DOI:** 10.1155/2019/1687345

**Published:** 2019-01-28

**Authors:** Ayman G. Mustafa, Ayssar A. Tashtoush, Othman A. Alshboul, Mohammed Z. Allouh, Ahmad A. Altarifi

**Affiliations:** ^1^College of Medicine, Qatar University, Doha, Qatar; ^2^Faculty of Medicine, Jordan University of Science and Technology, Irbid 22110, Jordan

## Abstract

This study was conducted to identify the morphometric features of the hard palate and to test the reliability of using palatal morphology in sex determination. Three hundred maxillary casts were collected from dental clinics in north Jordan. The age and gender of the patient and the serial number for each cast were recorded. The age range was 6 to 50 years old. A caliper was used to perform the following measurements: the length, width, and depth of the hard palate. In addition, the size, shape, and position of the incisive papilla were also determined. All measurements were done by a trained examiner who was able to perform the measurements in a reproducible manner. Statistical analysis showed that the mean palatal length, width, and depth, and size of dental papilla in both groups were the highest in males. The full logistic regression model including all the three predictors (length, width, and depth) indicated that the three parameters were significantly correlated with gender in the adult group. However, in the child group, only width and length were significantly (*p*=0.001, *p* > 0.042 respectively) correlated with gender. Regarding the shape and size of the incisive papilla, they were significantly different between males and females in both adult (*p* > 0.03) and child (*p*=0.001) groups. These findings might be potentially relevant to anthropological studies aiming at individual and/or sex identification. Moreover, the results might have clinical value in prosthodontics, especially in fabricating complete maxillary dentures for edentulous patients.

## 1. Introduction

The hard palate is the bony part of the palate comprising the anterior part of the palate [1]. It is an important part of the human skull that contributes to the separation of the oral and nasal cavities [2]. The hard palate is made of the palatine processes of the maxilla anteriorly and the horizontal plates of the palatine bones posteriorly. All of these bones meet at a cruciform system of sutures, which includes the median and transverse palatine sutures [3]. It is continuous with the intermaxillary suture between the upper central incisor teeth [4].

Typically, the tissues of the face develop from either side and fuse in the midline. This is correct for the development of the upper lip and the palate, which takes place within the first 30 to 60 days of pregnancy [5]. Qualitative sex differentiation using multiple bones has been discussed in the literature. Almost all elements of the human skeleton show some degree of sexual dimorphism [6–8]. Krogman and Iscan [7] described 14 indicators with an accuracy of 90% for helping with sex determination, and one of these indicators is the shape of the palate [8, 9]. Other recent reports continued to suggest that palatal morphology might be used for sex determination because it has many characteristic anatomical points, which allow easy and reproducible measurements [10, 11]. Further work in this area led researchers to propose that metric measurements of the palate might be reliable sex determinants [12–14].

The value of forensic dentistry in sex estimation and individual identification is beyond debate. This is related to the suggested stability and individuality of the dental and palatal structures including palatal rugae [4, 15]. The value of forensic dentistry in human identification is corroborated by the fact that palatal structures resist postmortem decomposition for several days and more so for the dental tissues [16, 17]. Moreover, palatal and dental structures are protected within the oral cavity which makes them resistant to damage by massive trauma and thermal insults. This makes sex estimation and individual identification using the morphometric features of the palate a convenient method of identification when there is massive tissue damage [16, 17].

The morphometric features of the palate are also of great importance in clinical dental sciences. The length, depth, and width of the palate have had considerable importance in orthodontic treatment planning and in the early diagnosis of oral disease [18]. Determination of the correct central incisor tooth position is necessary for fabricating conventional complete dentures or implant-supported prostheses that will successfully restore natural speech, aesthetics, and normal function [19, 20].

The horizontal relationship between the maxillary central incisors and the incisive papilla in dentate individuals is a guide to position the central incisor teeth as near to their original position as possible to restore the labial contour in edentulous patients [21]. The incisive papilla remains in a constant position after tooth and bone loss [22]. Moreover, several other reports continue to suggest that the incisive papilla is a reliable reference point for estimating the position of anterior teeth in edentulous patients during the setting of their teeth for dentures [19].

This study aimed at investigating the morphology of the hard palate in Jordanians and at generating normative values regarding the palatal length, depth, and width. It also aimed at establishing norms regarding the size and shape of the incisive papilla and the distance between the labial surface of central incisors and the incisive papilla in Jordanians and at further examining the reliability of the aforementioned parameters in sex determination.

## 2. Materials and Methods

This cross-sectional study was conducted in a random sample of Jordanian males and females. The study sample comprised 300 dental casts, which were divided into two groups: adults and children. The adult group included 150 casts from 66 males (44%) and 84 females (56%) aged 18–50 years old. The child group, on the contrary, included 150 casts from 75 males (50%) and 75 females (50%) aged 6–12 years old. A statistical power analysis showed that a sample size of at least 300 casts is needed to sufficiently represent the Jordanian population.

All subjects were fully dentate except for the third molars (with the exception of some subjects who had one or two missing teeth that did not affect the measurements). All subjects had no dental anomaly, no history of significant medical disease or trauma, no history of being subjected to any orthognathic surgery, no previous orthodontic treatment, and no extensive restorative procedure (i.e., crown and bridge work). Subjects suffering from severe malocclusion (e.g., open bite and crossbite) were excluded from the study. Moreover, all subjects were able to consent to receiving the procedures that were carried out (such as undergoing maxillary impressions). Data collection was performed by one trained examiner. Impressions of the upper jaw were made with silicon material, and casts were immediately poured in type II dental stone to minimize dimensional changes. Each of the fabricated casts was given a serial number. For each cast, the serial number and the age and gender of the patient were recorded and stored in the project files.

A digital caliper was used to measure palatal dimensions (depth and width), as well as the distance between the labial surface of the central incisors and the anterior border of the incisive papilla (CAIP) and the distance between the labial surface of the central incisors and the posterior border of the incisive papilla (CPIP), and the size of the incisive papillae (SIP) for each cast. The following measurements were made to the nearest millimeter.

The length was measured as the linear distance between the orale (the point at the anterior end of the incisive suture located between the sockets of the two central maxillary incisors) anteriorly to the midpoint of the linear distance between the distal surfaces of upper second molars in adult casts (mesial surfaces of the upper permanent first molar in the child casts) posteriorly.

The width was the distance between the inner borders of the sockets of the upper second molars in adult casts (upper permanent first molars in the child casts).

The depth was the distance between the inner border of the socket of the upper second molar (upper permanent first molar in the child casts) and the highest point of the palatal arch.

The borders of the incisive papilla were marked on each cast, and the shape of the incisive papilla was identified. The labial surface of the central incisor and the anterior and posterior borders of the incisive papilla were also marked on each cast. Then, the distance between the labial surface of the maxillary central incisor and the anterior border of incisive papilla (CAIP) and the distance between the labial surface of central incisor and the posterior border of incisive papilla (CPIP) were measured. This study was approved by the institutional review board at the Jordan University of Science and Technology.

### 2.1. Statistical Analysis

All data were numerically coded and manually transferred into a computer and exported to Statistical Package for the Social Sciences (SPSS) software. Using SPSS, descriptive statistics, independent-samples *t*-test, and binary logistic regression were calculated to perform comparisons between genders. The level of significance was set at *p* < 0.05.

The examiner who performed the measurements was trained on performing them. The accuracy of the trained examiner was tested by remeasuring 60 randomly selected casts (20%) by the same examiner. Moreover, variability and bias in the evaluation were assessed by remeasuring 60 randomly selected casts (20%) by another trained examiner who was blind to the study design and the cast origin. Original and repeated sets of measurements were analyzed for significance of differences, coefficient of reliability, and variance.

## 3. Results

### 3.1. Palatal Arch Dimensions for the Adult Group

The mean and standard deviation of the palatal arch dimensions, the width, length, and depth, were calculated, and they are reported in Table 1. The aforementioned parameters were compared between the adult males and females using Student's *t*-test. The palatal width, length, and depth were significantly (*p* < 0.05) higher in adult males than in adult females. Furthermore, the ability of each of these parameters to predict gender was tested using logistic regression. The results of binary logistic regression in the adult group showed that each of the three independent variables (palatal width, length, and depth) in the full logistic regression model was statistically significant, *x*_2_ = 157.37, *df* = 3, *N* = 150, and *p* < 0.05, indicating that the independent variables significantly predicted the outcome variable, gender. Length showed the most significant correlation with gender. This was evident by length having the odds ratio of 2.816.

### 3.2. Size of the Incisive Papillae, CAIP, and CPIP in the Adult Group

The mean values of the incisive papilla size, CAIP, and CPIP for both the genders are shown in Table 1. These measurements were compared between adult males and females using Student's *t*-test. Only the size of incisive papillae was significantly different between the two genders in the adult group.

### 3.3. Shapes of the Incisive Papilla in the Adult Group

Five shapes of the incisive papilla were seen (pear, cylindrical, flame, round, and double). Of these, the pear shape was the most common in Jordanian adult males (45.3%) and the flame shape was the most common in Jordanian adult females (52.8%) (Table 2).

### 3.4. Palatal Arch Dimensions for the Child Group

The mean and standard deviation of the palatal dimensions, width, length, and depth, were calculated, and they are reported in Table 3. The aforementioned parameters were compared between boys and girls using Student's *t*-test. Only palatal width and length were significantly (*p* < 0.05) higher in males than in females (Table 3). Furthermore, the ability of each of these parameters to predict gender was tested using logistic regression. The results of binary logistic regression in the child group showed that palatal width and length in the full logistic regression model were statistically significant, *x*_2_ = 67.347, *df* = 3, *N* = 150, and *p* < 0.05, indicating that palatal width and length significantly predicted the outcome variable, gender. Width showed the most significant correlation with gender. This was evident by it having an odds ratio of 1.708.

### 3.5. Size of the Incisive Papillae, CAIP, and CPIP in the Child Group

The mean values of the incisive papilla size, CAIP, and CPIP for both the genders in the child group are shown in Table 3. These measurements were compared between boys and girls using Student's *t*-test. Only the size of incisive papillae was significantly different between the two genders in the child group (Table 3).

### 3.6. Shapes of the Incisive Papillae in the Child Group

Five shapes of the incisive papilla were observed: pear, cylindrical, flame, round, and double. Of these, the cylindrical shape was the most common in Jordanian boys (37.3%) and the flame shape was the most common in Jordanian girls (78.6%) (Table 4).

## 4. Discussion

This is the first morphometric analysis study to investigate the hard palate in the Jordanian population. This study aimed at determining normative values regarding the palatal length, depth, and width in the Jordanian population. In addition, this is the first study to measure the size of the incisive papilla and the distance between the labial surface of central incisors and incisive papilla. The data provided by this study could be of particular value to the clinical practice of dentistry in Jordan. The results from this study might be incorporated into the process of designing and constructing dentures by prosthodontists for edentulous Jordanian patients. Moreover, data of the normal palatal size and size and shape of the incisive papilla might be relevant to the practice of oral medicine. For example, our data might be used as a baseline for studies investigating oral developmental abnormalities in the Jordanian population. In addition, these data might contribute to forensic science by providing potential sex determinants that might be utilized in human identification.

Though much has been published on geometric morphometric approaches to studying the human skeleton and the sexual dimorphism of its bones, the palatine bones themselves have received little attention [23, 24]. Nevertheless, the palatine bones intrigued some researchers who investigated their significance in various disciplines, including dentistry and developmental biology of the oral and nasal cavities [25]. This foundation of the potential values of the palate triggered our research group to investigate the morphometric features of the palate in Jordanians.

In our study, we reported that the palatal dimensions that reflect the palatal size were significantly higher in males than in females. This strongly suggests that the palatal dimensions and their overall size are sexually dimorphic. Our findings seem in-line with previously published reports in other populations using various study designs. For example, it was reported by a study performed on south Indian dry skulls that palatal dimensions were significantly higher in males and were hence sexually dimorphic [2]. Bigoni et al. who noted significant sex differences in the palatal dimensions in a European sample [23] reported comparable results. Moreover, Sumati and Phatak also found that the size of the palate, among five hard palate variables, was the best determinant of sex in a sample from the north Indian population [9]. Moreover, another research group in Brazil studied the widths of dental arches. They found that the maxillary arch width is larger in males than in females [18]. In addition to that, a systematic review was carried out to investigate the gender effect on palatal dimensions. The outcome of that review was in line with our results [26]. This harmony between our findings and previously published reports provides validity to our experimental design. It shows that the dental casts of the upper jaw reflect the anatomy of the palate as accurately as making direct measurements on dry skulls.

Regarding the sexual dimorphism of the palatal dimensions in the child group, our findings were also comparable with previous studies. For example, a previous study analyzed the palatal dimensions in 150 children and reported that all palatal measurements were larger in boys than in girls [27]. This indicates that sexual dimorphism of the palatal size is not dependent on whether or not puberty has been reached.

Several authors have investigated the horizontal relationship between the maxillary central incisors and incisive papilla. For example, Grave and Becker suggested that the labial surfaces of the maxillary central incisors should be 12-13 mm from of the posterior border of the papilla in the horizontal direction [19]. Other researchers provided different but comparable values regarding the distance between the incisive papilla and the anterior teeth in both dentate and denture-wearing individuals [20, 28]. Additionally, some researchers studied the anatomical location of the incisive papilla by measuring the distance between the center of the papilla and the labial surface of central incisors [29, 30]. The reason that these incisive papilla-related parameters received such immense attention by several research groups is because of their relevance to the clinical practice of prosthodontics.

During complete denture fabrication, restoring the natural dentolabial relations with the overall facial appearance is essential. This will guarantee successful treatment for edentulous patients not only in terms of function but also in terms of esthetics [31]. However, due to the continued alveolar bone resorption, it might be difficult to restore lost dental and oral tissues in the same approximate amounts and positions from which they were lost [28]. The data we provided in this study regarding the SIP, CAIP, and CPIP may help solving this problem by providing norms in the Jordanian population regarding the morphometric landmarks for arranging artificial upper anterior teeth during complete denture fabrication.

## 5. Conclusions

Some palatal dimensions display sexual dimorphism and can be used as predictors of sex. The sexual dimorphism of palatal dimensions is not only limited to adults but is also displayed in children. In addition to the palatal dimensions, the incisive papilla size and morphology display some degree of sexual dimorphism. Moreover, some of the studied parameters including the size of incisive papilla, the distance between the labial surface of the central incisors and the anterior border of the incisive papilla, and the distance between the labial surface of the central incisors and the posterior border of the incisive papilla might be helpful in improving the stability and esthetics of maxillary dentures for edentulous Jordanians. Having said that, it is noteworthy that the main limitation of our study is that it was restricted to people seeking dental treatment in north of Jordan. It would add to the value and validity of our results if we could perform a large-scale study aiming at establishing a national registry of the parameters we tested in our study.

## Figures and Tables

**Table 1:** Mean palatal arch dimensions, SIP, CAIP, and CPIP in Jordanian adults.

Variables	Male	Female	*t*-test
	Mean ± SD (mm)	Mean ± SD (mm)	*p* value
Length	43.91 ± 2.65	39.53 ± 2.73	0.000^*∗*^
Width	45.05 ± 2.47	40.23 ± 2.01	0.000^*∗*^
Depth	17.37 ± 2.05	15.69 ± 1.49	0.001^*∗*^
SIP	8.69 ± 1.37	4.16 ± 0.934	0.03^*∗*^
CAIP	4.74 ± 1.10	4.20 ± 0.96	0.496
CPIP	13.43 ± 1.42	11.71 ± 1.14	0.361

SIP, size of the incisive papilla; CAIP, distance between the labial surfaces of the central incisors and the anterior border of the incisive papilla; CPIP, distance between the labial surfaces of the central incisors and the posterior border of the incisive papilla. ^*∗*^Significant difference between males and females. Males and females were compared using Student's *t*-test.

**Table 2:** The percentages of different shapes of incisive papilla in Jordanian adults.

Shape of incisive papilla	Male (%)	Female (%)
Cylindrical	22.17	3.15
Flame	17.68	52.80
Pear	45.3	40.48
Round	14.85	2.38
Double	0.0	1.19

**Table 3:** Mean palatal arch dimensions, SIP, CAIP, and CPIP in Jordanian children.

Variables	Male	Female	*t*-test
	Mean ± SD (mm)	Mean ± SD (mm)	*p* value
Length	34.23 ± 2.98	32.20 ± 3.08	0.042^*∗*^
Width	39.39 ± 2.92	35.65 ± 2.28	0.000^*∗*^
Depth	12.22 ± 1.96	11.77 ± 1.65	0.671
SIP	7.00 ± 0.43	5.82 ± 0.36	0.000^*∗*^
CAIP	3.75 ± 0.65	3.20 ± 0.45	0.183
CPIP	10.9 ± 1.02	10.0 ± 0.99	0.165

SIP, size of the incisive papilla; CAIP, distance between the labial surfaces of the central incisors and the anterior border of the incisive papilla; CPIP, distance between the labial surfaces of the central incisors and the posterior border of the incisive papilla. ^*∗*^Significant difference between males and females. Males and females were compared using Student's *t*-test.

**Table 4:** The percentages of different shapes of incisive papilla in Jordanian children.

Shape of incisive papilla	Male (%)	Female (%)
Cylindrical	37.3	1.33
Flame	28	78.6
Pear	33.37	17.4
Double	1.33	2.67

## Data Availability

The data used to support the findings of this study may be released upon application to the Institutional Review Board at the Jordan University of Science and Technology, who can be contacted via medicine@just.edu.jo.

## References

[1] Jotaial B., Patel S., Patel S., Patel P., Patel S., Patel K. (2013). Morphometric analysis of hard palate. *International Journal of Research in Medical Sciences*.

[2] D’Souza A., Mamatha H., Jyothi N. (2012). Morphometric analysis of hard palate in South Indian skulls. *Biomedical Research*.

[3] Varalakshmi K. L., Sangeeta M., Shilpa N., Arunashri A. (2015). An osteological study of morphometry of hard palate and its importance. *International Journal of Research in medical sciences*.

[4] Mustafa A. G., Allouh M., Tarawneh I., Alrbata R. (2013). Morphometric analysis of palatal rugae among Jordanians: further evidence of worldwide palatal rugae individuality. *Australian Journal of Forensic Sciences*.

[5] Standring S. (2015). *Gray’s Anatomy*.

[6] Suazo Galdames I. C., Zavando Matamala D. A., Luiz Smith R. (2008). Accuracy of palate shape as sex indicator in human skull with maxillary teeth loss. *International Journal of Morphology*.

[7] Krogman W. M., Iscan M. Y. (1986). *The Human Skeleton in Forensic Medicine*.

[8] Manjunath T. H., Kuppast N. C., Shahina, Umesh S. R., Iddalgave S. (2014). Identification of gender from dimensions of palate. *Medico-Legal Update*.

[9] Sumati P. V. V. G., Phatak A. (2012). Determination of sex from hard palate by discriminant function analysis. *International Journal of Basic and Applied Medical Sciences ISSN*.

[10] Babaji P., Jalal S. A., Kamalaksharappa S. K. (2018). Evaluation of palatal rugae pattern in identification and sex determination in Indian children. *Pesquisa Brasileira em Odontopediatria e Clínica Integrada*.

[11] Tomaszewska I. M., Frączek P., Gomulska M. (2014). Sex determination based on the analysis of a contemporary Polish population’s palatine bones: a computed tomography study of 1,200 patients. *Folia Morphologica*.

[12] Iscan M. Y., Helmer R. P. (1993). *Forensic Analysis of the Skull: Craniofacial Analysis, Reconstruction, and Identification*.

[13] Jacob M., Bindhu S., Avadhani R. (2016). Sex determination from hard palate measurements using palatine index with reference to its clinical implications. *Indian Journal of Clinical Anatomy and Physiology*.

[14] Shalaby S. A. (2015). Morphometric analysis of hard palate in Egyptian skulls. *Revista Argentina de Anatomía Clínica*.

[15] Mustafa A. G., Allouh M. Z., Alshehab R. M. (2015). Morphological changes in palatal rugae patterns following orthodontic treatment. *Journal of Forensic and Legal Medicine*.

[16] Caldas I. M., Magalhaes T., Afonso A. (2007). Establishing identity using cheiloscopy and palatoscopy. *Forensic Science International*.

[17] Sweet D., DiZinno J. A. (1996). Personal identification through dental evidence–tooth fragments to DNA. *Journal of the California Dental Association*.

[18] Louly F., Nouer P. R., Janson G., Pinzan A. (2011). Dental arch dimensions in the mixed dentition: a study of Brazilian children from 9 to 12 years of age. *Journal of Applied Oral Science*.

[19] Grave A. M. H., Becker P. J. (1987). Evaluation of the incisive papilla as a guide to anterior tooth position. *Journal of Prosthetic Dentistry*.

[20] Park Y.-S., Lee S.-P., Paik K.-S. (2007). The three-dimensional relationship on a virtual model between the maxillary anterior teeth and incisive papilla. *Journal of Prosthetic Dentistry*.

[21] Solomon E. G. R., Arunachalam K. S. (2012). The incisive papilla: a significant landmark in prosthodontics. *Journal of Indian Prosthodontic Society*.

[22] Soo-Yeon S. (2016). Correlation between the size of the incisive papilla and the distance from the incisive papilla to the maxillary anterior teeth. *Journal of Dental Sciences*.

[23] Bigoni L., Velemínská J., Brůžek J. (2010). Three-dimensional geometric morphometric analysis of cranio-facial sexual dimorphism in a Central European sample of known sex. *Homo*.

[24] Chovalopoulou M.-E., Valakos E. D., Manolis S. K. (2013). Sex determination by three-dimensional geometric morphometrics of the palate and cranial base. *Anthropologischer Anzeiger*.

[25] Ambrosio E. C. P., Sforza C., De Menezes M. (2018). Longitudinal morphometric analysis of dental arch of children with cleft lip and palate: 3D stereophotogrammetry study. *Oral Surgery, Oral Medicine, Oral Pathology and Oral Radiology*.

[26] Berwig L. C., Marquezan M., Milanesi J. d. M., Montenegro M. M., Ardenghi T. M., Toniolo da Silva A. M. (2018). O gênero e a idade influenciam as dimensões do palato duro? Revisão sistemática da literatura. *CoDAS*.

[27] Hung-Huey T., Ching-Ting T. (2004). Morphology of the palatal vault of primary dentition in transverse view. *Angle Orthodontist*.

[28] Isa Z. M., Abdulhadi L. M. (2012). Relationship of maxillary incisors in complete dentures to the incisive papilla. *Journal of Oral Science*.

[29] Amin W. M., Taha S. T., Al-Tarawneh S. K., Saleh M. W., Ghzawi A. (2008). The relationships of the maxillary central incisors and canines to the incisive papilla in Jordanians. *Journal of Contemporary Dental Practice*.

[30] Shrestha S., Joshi S. P., Yadav S. K. (2016). Relationship of incisive papilla to maxillary incisors and canines. *Journal of Contemporary Dental Practice*.

[31] Re D., Augusti D., Torquati Gritti U., Riva G., Augusti G. (2012). Esthetics in the edentulous: clinical steps for recovering of maxillary anterior teeth harmony. *Minerva Stomatologica*.

